# Application of FRET Biosensors in Mechanobiology and Mechanopharmacological Screening

**DOI:** 10.3389/fbioe.2020.595497

**Published:** 2020-11-09

**Authors:** Longwei Liu, Fangchao He, Yiyan Yu, Yingxiao Wang

**Affiliations:** Department of Bioengineering, Institute of Engineering in Medicine, University of California, San Diego, La Jolla, CA, United States

**Keywords:** FRET, mechanotransduction, cell-ECM interaction, live-cell imaging, drug-screening

## Abstract

Extensive studies have shown that cells can sense and modulate the biomechanical properties of the ECM within their resident microenvironment. Thus, targeting the mechanotransduction signaling pathways provides a promising way for disease intervention. However, how cells perceive these mechanical cues of the microenvironment and transduce them into biochemical signals remains to be answered. Förster or fluorescence resonance energy transfer (FRET) based biosensors are a powerful tool that can be used in live-cell mechanotransduction imaging and mechanopharmacological drug screening. In this review, we will first introduce FRET principle and FRET biosensors, and then, recent advances on the integration of FRET biosensors and mechanobiology in normal and pathophysiological conditions will be discussed. Furthermore, we will summarize the current applications and limitations of FRET biosensors in high-throughput drug screening and the future improvement of FRET biosensors. In summary, FRET biosensors have provided a powerful tool for mechanobiology studies to advance our understanding of how cells and matrices interact, and the mechanopharmacological screening for disease intervention.

## Introduction

During the past decades, it has been well-acknowledged that, the mechanical microenvironment plays a crucial role in regulating cellular functions, together with biochemical signals (Wang et al., 2009). The dysfunction of mechanical microenvironments such as shear stress (Zhou et al., 2014), ECM properties (Bonnans et al., 2014) (e.g., stiffness, degradability, remodeling capacity, porosity, topography, internal strain, and intracellular mechanotransduction) (Humphrey et al., 2014) could contribute to the pathogenesis of multiple diseases, including but not limited to atherosclerosis (Chiu and Chien, 2011), fibrosis (Liu et al., 2020), and cancer (Branco da Cunha et al., 2016). As such, elucidating how the mechanical microenvironment influences cell behavior in physiological and pathological conditions, and the successful identification of mechanotransduction-modulating small molecules would pave the way for next-generation disease-intervention strategies. However, conventional biochemical assays (e.g., western blot, immunostaining) are limited in spatiotemporal resolutions in elucidating instant (usually happens within several seconds) and dynamic mechanotransduction processes (Wang and Wang, 2009; Wang et al., 2009; Liu et al., 2020). Imaging techniques together with genetically encoded FRET biosensors can be a powerful approach for dynamically tracking signal transduction in live cells or tissues with high spatiotemporal resolutions, thus enabling the investigation of mechanotransduction during cell-microenvironment and cell-cell interactions. Here in this review, we will briefly introduce FRET technology, the application of FRET biosensors in the studies of mechanobiology, and for high-throughput drug screening. Finally, the future perspective of FRET biosensors and their applications will be discussed.

## FRET and FRET Biosensors

Förster or fluorescence resonance energy transfer biosensor allows studying the dynamics of signaling molecule activities in living cells. It has been increasingly applied in the mechanobiological study due to its superiority in ratiometric fluorescence readout, high signal-to-noise ratio, and high spatiotemporal resolution compared to biochemical assays (Wang and Wang, 2009). In this section, the FRET principle and the classification of FRET biosensor will be introduced.

### FRET

**FIGURE 1:**
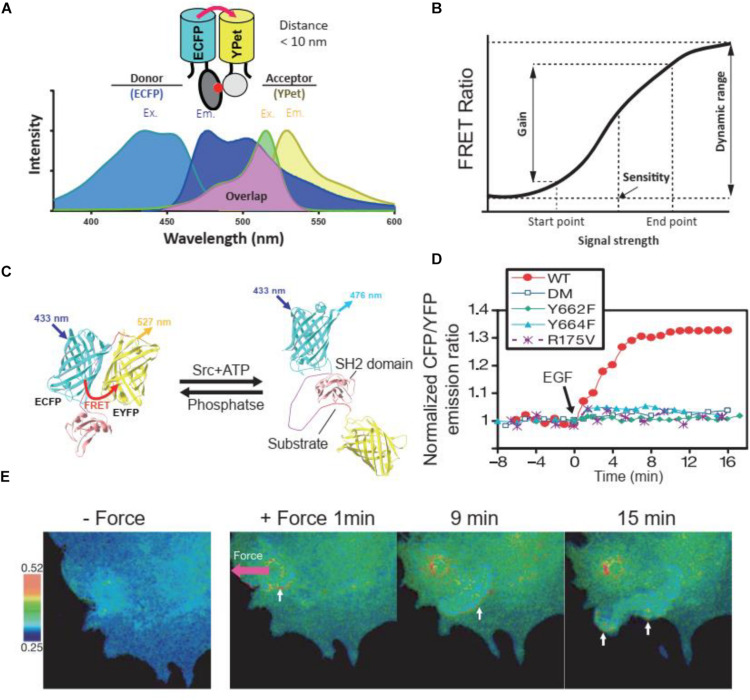
FRET principles and FRET biosensors. **(A)** Excitation (Ex.) and Emission (Em.) wavelength of ECFP and YPet, as a FRET donor and receptor, respectively. The emission wavelength of ECFP overlaps with the excitation wavelength of YPet, enables the energy transfer between donor and receptor when their distance is less than 10 nm. **(B)** The terminology used to describe and quantify the performance of FRET biosensors. The figure was modified based on previous publication (Komatsu et al., 2011). **(C)** The cartoon illustrates the FRET change of a Src kinase biosensor at the presence of Src kinase or phosphatase. **(D)** Time course quantification of the Src FRET biosensor and its loss of function mutants in response to EGF stimulation in HeLa cells. Y662F and Y664F, single loss-of-function mutations in the substrate of the biosensor; DM, double mutation (Y662F and Y664F); R175V, loss-of-function mutation in SH2 domain. **(E)** FRET responses of a cell with clear directional wave propagation away from the site of mechanical stimulation. In these images, a pulling force was applied via laser tweezers on a bead coated with fibronectin on the cell membrane. **(C–E)** were obtained from a previous publication (Wang et al., 2005).

Förster or fluorescence resonance energy transfer is a phenomenon of non-radiative energy transfer through dipole-dipole coupling between two fluorophores, in which the transferred emission energy from the donor triggers fluorescence emission of the acceptor positioned typically within 1 to 10 nanometers (nm) (Forster, 1946; Clegg, 1995; Sekar and Periasamy, 2003; Wang and Wang, 2009; Ray et al., 2014; Bajar et al., 2016). The efficiency of FRET (*E*) depends on the spectral overlap between the emission spectrum of the donor and the excitation spectrum of the acceptor (*J*), the distance between the two fluorophores (*r*), and the relative orientation between the dipoles of the donor and the acceptor represented by the orientation factor (κ^2^) (Miyawaki, 2003; Wang and Wang, 2009; Figure 1A). According to Förster’s Theory, *E* is related to the sixth power of *r* normalized by the Förster radius (*R*_0_) (see Eq. 1), where *R*_0_ is defined as the separation at 50% energy transfer (Förster, 1948). *R*_0_ is dependent upon κ^2^, *J*, the quantum yield of the donor (*Q*_D_), and the index of refraction of the transfer medium (*n*) (see Eq. 2) (Stryer, 1978). Specifically, *J* is the normalized integral of the product of donor intensity (*F*_D_), molar absorbance of the acceptor (*E*_A_), and the fourth power of wavelength (λ^4^) over the entire spectrum (see Eq. 3). κ^2^ is given by the angle between transition moments of the FRET pair (α) as well as the angles between the line connecting two fluorophores and the donor and acceptor moments (β and γ), respectively, (see Eq. 4). Assuming a complete and random orientation, κ^2^ of the freely rotated fluorophores is simplified to 2/3, from which the apparent distance between two fluorophores (*r’*) is calculated. The actual distance *r* can be estimated from κ^2^ and *r’* (see Eq. 5). Simply, the larger the spectral overlap integral between donor emission and acceptor excitation, the closer distance between the donor and receptor, the higher the FRET efficiency (Stryer, 1978; Hochreiter et al., 2015).

(1) $$J=\frac{\int F_{D}\left(\lambda \right)E_{A}\left(\lambda \right)\lambda^{4}\text{d}\lambda }{\int F_{D}\left(\lambda \right)\text{d}\lambda }=\int \bar{F_{D}}\left(\lambda \right)E_{A}\left(\lambda \right)\lambda^{4}\text{d}\lambda$$

(2) $$R_{0}^{6}=\left(8.79\times 10^{-25}{\text{cm}}^{6}\right)n^{-4}Q_{D}k^{2}J$$

(3) $$E=\frac{1}{1+{\left(\frac{r}{R_{o}}\right)}^{6}}$$

(4) $$k^{2}={\left(\text{cos}\alpha -3\cos\beta \cos\gamma \right)}^{2}$$

(5) $$r={\left(1.5k^{2}\right)}^{\frac{1}{2r^{\prime }}}$$

Due to its sensitivity to intermolecular distance and orientation, FRET biosensors have been designed to precisely measure those quantities between two sites of molecules through the detection and quantification of emission signals of FRET pairs (Hochreiter et al., 2015). Although different small organic dyes, quantum dots (QDs), and fluorescent proteins (FPs) have been used as FRET pairs in FRET biosensors, the genetically encoded FPs were more wildly used in live-cell FRET imaging due to the superiority in compatibility and stability in a stable cell line or *in vivo* via genetic engineering (Bajar et al., 2016). Among the FPs, cyan FP-yellow FP (CFP-YFP) pair is commonly used as FRET pair due to the significant overlap between the emission spectrum of CFP and the excitation spectrum of YFP (Shimozono and Miyawaki, 2008; Wang and Wang, 2009). Derived from that pair, ECFP and YPet were demonstrated to have significantly improved sensitivity as a FRET pair in biosensors for single-cell imaging (Ouyang et al., 2008b; Seong et al., 2009). Besides that, green FPs (GFPs), red FPs (RFPs), far-red FPs (FFPs), and infrared FPs (IFPs) could also be used as FRET pairs in a biosensor as summarized previously (Bajar et al., 2016). In addition to a FRET pair, a typical FRET biosensor also contains a ligand and a sensor domain (Aoki et al., 2012), the binding of which determines the conformation of the biosensor and the proximity of the FRET pair (Wang and Wang, 2009). As such, the binding between the ligand and the sensor domain in a FRET biosensor can be designed to detect and measure a specific cellular activity (Stryer, 1978; Wang and Wang, 2009).

The dynamic range, gain, and sensitivity are the interrelated technical terms describing the performance of FRET biosensors (Komatsu et al., 2011; Figure 1B). When cells with biosensor excited at the optimal wavelength in the excitation spectrum of the donor, the emission ratio of acceptor over donor fluorescence intensity, called the FRET ratio, is used to represent the states of FRET biosensors. FRET ratio and images were wildly used to illustrate the dynamic signaling events in cells. For example, when the substrate in the Src biosensor was phosphorylated by endogenous Src kinase after either chemical or mechanical stimulation, the FRET efficacy would change (Figures 1C–E), thus leading to the change of FRET ratio. Through FRET ratio (CFP/YFP in this case) calculation, we could quantify (Figure 1D) or visualize (Figure 1E) the dynamic change of Src kinase activity in cells. The range of the FRET ratio at all states of the biosensor is defined as the dynamic range. A high dynamic range level reflects a high efficiency and good quality of the FRET biosensor (Kolossov et al., 2011). Gain is the percentage change of the FRET ratio after cellular activity change and was used to quantify the change of FRET ratio before and after stimulation or inhibition. Sensitivity is defined as the concentration of the stimulants that increase the FRET ratio to 50% of the dynamic range (Komatsu et al., 2011). The measurements of these parameters are utilized to evaluate FRET biosensors and are used as the criteria in biosensor optimization (Komatsu et al., 2011).

### Classification of FRET Biosensors

**FIGURE 2:**
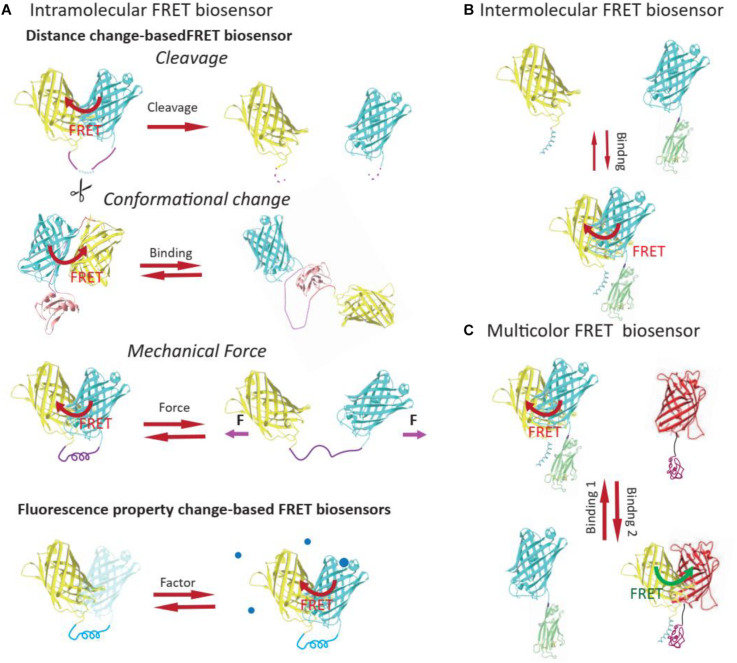
Classification of FRET biosensors. Based on whether the donor and the acceptor are on the same molecule, FRET biosensors can be classified into intramolecular **(A)** or intermolecular FRET biosensors **(B)**. The intramolecular FRET biosensor could be further classified into distance change-based FRET biosensor or FP property change-based FRET biosensors, based on the mechanism of FRET ratio change. **(C)** Multicolor FRET biosensors usually contain more than two FPs and could be used for imaging of different cellular events in the same live cell.

Förster or fluorescence resonance energy transfer biosensors can be classified as intramolecular (unimolecular) or intermolecular (bimolecular) depending on whether the donor and the acceptor are on the same molecule (Miyawaki, 2003; Bajar et al., 2016; Figures 2A,B). Intramolecular FRET biosensors can be further categorized into two main types on the basis of how the FRET ratio is altered upon detection of a chemical signal: distance change-based and fluorescence property change-based. Furthermore, based on how the distances (and orientations) between donors and acceptors are altered, distance change-based FRET biosensors can be classified into three major types: cleavage-based, conformational change-based, and mechanical force-based (Figure 2A), which have been wildly applied in mechanobiology studies and were summarized in an earlier publication (Hochreiter et al., 2015). Fluorescence property change-based FRET biosensors report FRET change based on the altered fluorescent ability of fluorophores as a result of the chemical environment, such as pH and oxidation. Besides, while most of the FRET biosensors have two FPs as FRET pair, multicolor FRET biosensors have been developed recently for imaging of different cellular processes in the same cell (Figure 2C).

#### Distance Change-Based FRET Biosensors

The FRET efficiency is highly sensitive to the distance change between donor and receptor, which could be induced by (1) cleavage of sensing domain and the consequent diffusion of donor and receptor, (2) the conformational change of FRET pairs, and (3) mechanical tension.

##### Cleavage-based FRET biosensors

Cleavage-based FRET biosensors consist of donors and acceptors closely connected by short linkers, which can be cleaved by specific enzymes (Hochreiter et al., 2015). In the uncleaved form, the energy transfer from the donors in proximity results in strong fluorescent signal emission by the acceptors, but once the linkers are recognized and cleaved, the donors and acceptors are separated by diffusion to cause FRET reduction. Currently, cleavage-based biosensors have been applied to the detection of apoptosis (Bozza et al., 2014), necroptosis (Sipieter et al., 2014), autophagy (Li et al., 2012), and extracellular matrix (ECM) remodeling (Chung et al., 2015). In mechanobiology, the remodeling of ECM could be visualized by the cleavage-based matrix metalloproteinases (MMPs) biosensors.

##### Conformational change-based FRET biosensors

In conformational change-based FRET biosensors, the alteration of distances between donors and acceptors is triggered via the conformational changes of proteins during biological processes (Wang et al., 2005). Usually, this type of biosensors utilizes binding domains that detect post-translational modification, including but not limited to phosphorylation, acetylation, and methylation and cellular reactions to environmental changes. Hence, they are widely used in the investigations of signal transduction, mechanotransduction, metabolite quantification, drug efficacy, and T cell interaction, in which specific proteins are integrated into FRET biosensors to track their activation and subcellular localization. In addition to the Src biosensor, which has been applied to visualize the chemically (Figure 1D) or mechanically (Figure 1E) induced Src kinase activation, a Lck kinase FRET biosensor with a substrate peptide (MDTSVFESPYSDPEE) derived from Zap70 kinase, namely ZapLck, has been used to investigate the biophysical basis underlying dynamic Lck activation in T cells (Wan et al., 2019).

##### Mechanical Force-based FRET biosensors

Mechanical forces applied to cells and proteins will alter the conformation and output signal of FRET biosensors. Usually, the donor and acceptor in the mechanical force-based FRET biosensors were connected by a flexible linker, and the distance between donor and receptor will increase after force application, as a way to directly detect and quantify the force-induced effects. Therefore, mechanical force-based FRET biosensors have been used for mechanical testing of strain in the ECM, between neighboring cells (Kim et al., 2015), intracellular tension, cell-ECM adhesion, cell traction force, and fluid shear stress. We will present a detailed discussion of the application of mechanical force-based FRET biosensors in mechanobiology in the following section “Mechanical Force FRET Biosensor.”

#### Fluorescence Property Change-Based FRET Biosensors

The fluorescent ability of some fluorophores may change under some specific conditions, such as pH change. For example, utilizing pH-sensitive FP variants, FRET reporters were developed for the detection of pH in different cellular compartments and extracellular microenvironments (Awaji et al., 2001; Urra et al., 2008). Ever since, several fluorescence property change-based FRET sensors have been developed to monitor pH changes of a broad range (Rupprecht et al., 2017).

#### Multicolor FRET Pairs

Compared with two-color FRET biosensors, three-color FRET biosensors consist of three fluorophores, in which the first fluorophore transfers its energy to the second fluorophore, which could serve as a donor for energy transfer to the third one, triggering the emission signal of the third fluorophore (Kienzler et al., 2011). Another schematic of three-color FRET biosensor is that both of the first two fluorophores transfer energy to the third one, while the first fluorophore also transfers energy to the second one (Lee et al., 2010; Sun et al., 2010; Uhm et al., 2018). Three-color FRET biosensors are commonly applied to advanced analysis of DNA and proteins, such as tracking the conformational changes of proteins in live cells (Kienzler et al., 2011; Wallrabe et al., 2015). Since there are more parameters involved in the measurements, one challenge of using three-color FRET biosensors is the relatively complicated interpretation and analysis of data (Wallrabe et al., 2015).

## FRET Biosensor and Mechanobiology

Cells are embedded in 3D ECM *in vivo*, and the mechanical factors from the microenvironment can influence cellular functions. FRET biosensors have been successfully applied in studying mechanobiology, i.e., how these cells perceive environmental mechanical cues. In this part, we will summarize the current progress of FRET biosensors and their application in visualizing and quantifying the dynamic cell-environment interaction and intracellular mechanotransduction.

### Application of FRET Biosensors in Visualizing Cell-Environment Interaction

**FIGURE 3:**
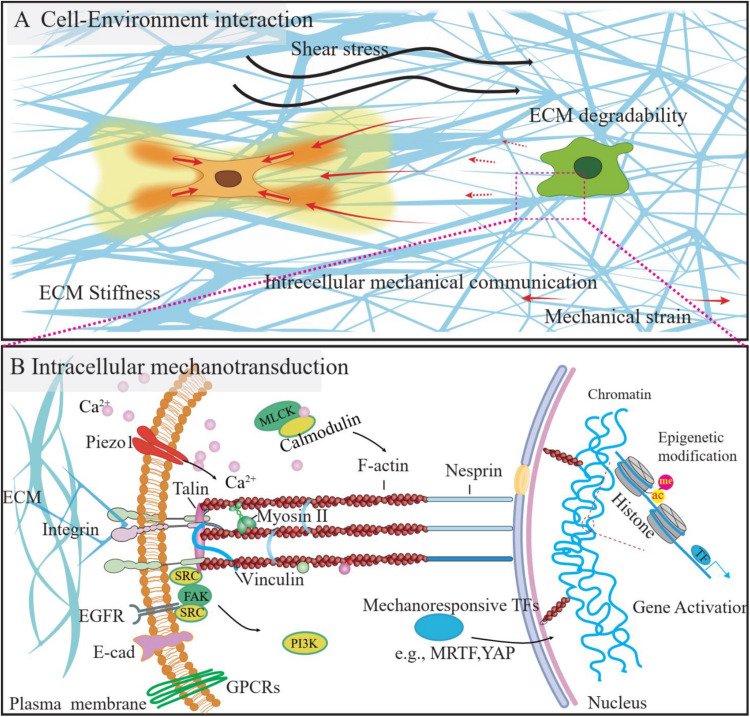
The mechanical microenvironment and mechanotransduction process of the cells. **(A)** The cells reside in the 3D microenvironment, the mechanical stimulation from the microenvironment, such as the shear stress, change of ECM properties (e.g., stiffness, degradability) could influence the cell function. Besides, the ECM fiber could be used as a media to transmit the mechanical strain from the neighboring cells, as a way of intercellular communications. **(B)** Cells could sense and respond to mechanical stimuli from the environment by converting them to intracellular biochemical signals, which process is called mechanotransduction. Several signaling pathways were identified to be involved in this process and could be visualized by using FRET biosensors, as listed above.

Abundant studies demonstrate that cells can sense and respond to the environment’s mechanical properties, such as stiffness (Engler et al., 2006), ECM degradability (Trappmann et al., 2017), viscoelasticity (Chaudhuri et al., 2015), remodeling capacity (Baker et al., 2015; Davidson et al., 2019), porosity (Jiang et al., 2019), topography (Cutiongco et al., 2020), and so forth. As a way of intercellular communication, cells can respond to the paratensile signal exerted by neighboring cells (Liu et al., 2020). In addition, the frictional force generated by blood flow (fluid shear stress, FSS) in the endothelium, can modulate cellular functions (Zhou et al., 2014). FRET biosensors have successfully been applied in visualizing and quantifying extracellular, intercellular, and intracellular mechanical forces (Figure 3A) and the relevant biological events.

#### Mechanical Force FRET Biosensor

When external forces, either from the non-cell environment components or neighboring cells, were applied to cells, these forces were transmitted from the ECM to the cell, which have been regarded as the “outside-in” signal. Fibronectin (FN) FRET biosensors were developed and successfully applied to visualize and quantify these extracellular force signals. FN is a major class of ECM molecules and could be reversibly unfolded when stretched. Upon unfolding, exposure of cryptic sites on FN can not only increase the tensile strength of FN and mediate fibrillogenesis, but also alter its binding preferences to different integrin isotypes (Vogel, 2018). The application of FRET to the detection of FN conformational states involved the labeling of FN with multiple donor and acceptor fluorophores and demonstrated an extended state of cell-bound FN prior to fibrillogenesis *in situ* (Baneyx et al., 2001). A subsequent study utilizing the fluorophore-labeled FRET-FN probe found that the partial unfolding state of FN requires cytoskeletal tension (Baneyx et al., 2002). A FRET ratio versus strain curve was constructed through an *in vitro* assay to probe FN conformation in cell cultures with a high spatial resolution (Little et al., 2008).

Förster or fluorescence resonance energy transfer-FN has also been employed to visualize FN conformational changes upon adsorption to the surface of materials with varying hydrophobicity, which revealed more extended FN conformation and better cell attachment on hydrophilic materials (Baugh and Vogel, 2004). Applying this FRET-FN probe, more recent research revealed the co-localization of collagen I and relaxed FN (Kubow et al., 2015), transformation of FN-coated collagen during force-mediated 3D tissue remodeling (Legant et al., 2012), spatial variations of FN conformation in a 3D *in vitro* model (Foolen et al., 2016), and the upregulation of low-tension FN by Ficoll crowding that promotes collagen fibrillogenesis (Graham et al., 2019). Additionally, FRET-FN has been used to investigate FN conformation in cancer malignancy, which revealed matrix stiffening and pro-angiogenic effect of the early interaction between unfolded FN and collagen near breast cancer cells (Wang et al., 2015, 2017). A domain-specific FP-based FRET probe was also developed to elucidate the conformation of III_1_ and III_2_ domains of FN (Karuri et al., 2009). However, the usage of FP-based FN FRET tension reporters has been cautioned, as GFP has been shown to reduce its emission after stretching (Saeger et al., 2012).

Cells also could generate cell traction force itself during the migration, or as a response to tissue stiffness, and consequently, deform the ECM or affect the neighboring cells (Liu et al., 2017). This force has been referred to trigger the “inside-out” signaling. FRET biosensors have been applied to visualize these kinds of cell traction and force reactions. At the cellular level, these mechanical forces were generated and mediated by ECM receptors, cytoskeletal filaments, and their related proteins (Wang, 2010). For example, vinculin plays a crucial role in force transmission and focal adhesion formation, and its activity is closely associated with talin. A vinculin tension sensor (VTS) was developed by inserting a pair of a monomeric teal fluorescent protein (mTFP1) and Venus (A206K) connected by an elastic domain between vinculin head and tail, in which the FRET ratio directly reports the distance between two fluorophores and indirectly measures the tension across vinculin (Grashoff et al., 2010). The VTS demonstrated independent regulation of vinculin recruitment and force transmission, with high tension across vinculin located in the protruding regions, low tension across vinculin in the retracting regions during cell migration, albeit vinculin is required for FA stabilization under force (Grashoff et al., 2010). Talin is an adhesion plaque protein that connects actin-based cytoskeleton to integrin-mediated ECM adhesion (Yao et al., 2016) and cadherin-based cell-cell junctions (Leckband and Rooij, 2014). Similar to vinculin, talin also contains an integrin-binding head domain and an actin-binding rod domain (Kumar et al., 2016). Hence, a talin tension sensor (talin TS) adopted a similar structure to VTS and reported higher talin tension in the peripheral of FA and vinculin-induced talin tension (Kumar et al., 2016). Similarly, the insertion of mTFP-elastic linker-mEYFP between the transmembrane domain and the β-catenin binding domains of E-cadherin gave rise to an E-cadherin tension FRET sensor, which demonstrated the constitutive cytoskeleton-to-membrane mechanotransduction function of E-cadherin throughout the epithelial cell surface (Borghi et al., 2012). However, a more recent study using a similar construct of the E-cadherin FRET tension sensor revealed several technical issues in force measuring in *Drosophila* tissues (Eder et al., 2017), highlighting the difficulties in application of FRET biosensor *in vivo* and the urgent need in further optimization of FRET biosensors.

#### ECM Degradation FRET Biosensor

Matrix metalloproteinases (MMPs) are membrane-bound or secreted enzymes that degrade ECM (Page-McCaw et al., 2007). In addition to ECM degradation, the functions of MMPs include clearing paths and providing cues for cell migration, cleaving intercellular junctions and basement membranes, and regulating signaling molecules with diverse consequences (Page-McCaw et al., 2007).

Detection of MMP catalytic activities requires cleavage-based FRET biosensors with an MMP substrate sequence sandwiched by two fluorophores. The most studied MMP using FRET reporters is the membrane type 1 matrix metalloproteinase (MT1-MMP or MMP-14). Constructed with a substrate sensitive to MT1-MMP, the first MT1-MMP FRET sensor observed epidermal growth factor (EGF)-induced, cytoskeleton-dependent MT1-MMP activity at the leading edge of migrating cancer cells, which can be abolished by tissue inhibitor of metalloproteinase-2 (TIMP-2) (Ouyang et al., 2008a). Subsequently, simultaneous imaging of MT1-MMP and Src activities utilizing two membrane-anchored FRET biosensors of different color schemes (orange/red and cyan/yellow) revealed spatiotemporal differences between MT1-MMP and Src activation upon EGF stimulation (Ouyang et al., 2010). Due to the lack of specificity of the MT1-MMP substrate sequence utilized in previous studies, directed evolution revealed a consensus sequence that enhances cleavage rates (Jabaiah and Daugherty, 2011). Furthermore, a quantum dot-based FRET biosensor was developed to bypass gene delivery and monitor membrane-bound MT1-MMP activity (Chung et al., 2015). Recently, a FRET probe linked with binding domains of MT1-MMP further enhanced the specificity of MT1-MMP activity visualization in tumors (Ji et al., 2020).

Similar cleavage-based FRET reporters have been constructed for MMP-2 (Yang et al., 2007; Lee et al., 2020), MMP-9 (Stawarski et al., 2014), MMP-11 (Meyer and Rademann, 2012), MMP-12 (Lim et al., 2014), and MMP-13 (Lim et al., 2014; Duro-Castano et al., 2018). Interestingly, a dual-FRET biosensor was developed for simultaneous imaging of MMP-2 and caspase-3 activities, in which the sequential cleavage by MMP-2 and caspase-3 increased the donor emission intensity in a stepwise fashion (Li et al., 2015).

In addition to cleavage-based FRET sensors, bimolecular FRET biosensors have been employed to investigate the dimerization of MT1-MMP and the interaction between CD44 and MT1-MMP during cell invasion (Marrero-Diaz et al., 2009; Itoh et al., 2011), as well as the association of CD151 and MT1-MMP in angiogenesis (Yañez-Mó et al., 2008).

#### Sheer Stress FRET Biosensor

Cadherins are a class of cell adhesion molecules (CAMs) and distinguish themselves from integrins for their calcium dependence. In particular, vascular endothelial cadherin (VE-cadherin) and epithelial cadherin (E-cadherin) play crucial roles in the mechanotransduction of shear stress. VE-cadherin, along with platelet endothelial cell adhesion molecule 1 (PECAM-1) and vascular endothelial growth factor receptor 2 (VEGFR2), constitutes a mechanosensory complex and each generates differential mechanical responses to FSS (Tzima et al., 2005; Conway and Schwartz, 2015). Shear stress can also induce an oscillatory movement in epithelial cells mediated by the linkage of E-cadherin and actomyosin cytoskeleton (Sadeghipour et al., 2018).

A FRET biosensor with the insertion of mTFP1-elastic linker-Venus between the p120 catenin and the β-catenin binding domains of VE-cadherin revealed that junctional VE-cadherin tension decreased in response to FSS from blood flow, whereas a PECAM-1 tension sensor with a similar design reported an increase in PECAM-1 tension in cell-cell junctions through vimentin association (Conway et al., 2013). A further study applying this VE-cadherin tension sensor reported similar results during the maturation of arterial junctions in zebrafish (Lagendijk et al., 2017). Interestingly, this study found no change in VE-cadherin tension after a complete loss of blood flow, which may suggest a lack of regulation by flow after junction maturation (Lagendijk et al., 2017).

### Application of FRET Biosensors in Investigating Intracellular Mechanotransduction

After perceiving mechanical stimulations, cells will convert biomechanical signals into biochemical signals and elicit cellular responses via the mechanotransduction process. Several intracellular signaling pathways were identified to be involved in the mechanotransduction of cells, including but not limited to GPCR signaling, kinase signaling, ECM receptor signaling, and calcium signaling, as summarized in the following part. The activities of these signaling pathways will finally lead to gene expression and cell phenotype change, either by influencing the mechanoresponsive transcription factors [e.g., MRTF (Finch-Edmondson and Sudol, 2016) and YAP (Low et al., 2014)] or exerting epigenetic modifications. Accordingly, several FRET biosensors were developed and successfully applied in intracellular mechanotransduction studies (Figure 3B).

#### Calcium FRET Biosensor

For two decades, FRET biosensors have significantly contributed to the investigation of the role of Ca^2+^ in intracellular mechanotransduction pathways. There are two major series of Ca^2+^ FRET reporters, calmodulin (CaM)-based (Cameleons) and troponin-based sensors (TN Ca^2+^ sensors). Miyawaki et al. (1997) first applied FRET to a Ca^2+^ biosensor, named Cameleon, comprised of an FP pair derived from GFP, calmodulin (CaM), and a CaM-binding peptide M13. Upon binding of four Ca^2+^ cations, CaM wraps around M13, triggering the conformational change of the biosensor and bringing two fluorophores in proximity (Miyawaki et al., 1997). Ever since the development of the first Cameleon, mutagenesis, rational design and circular permutation have been applied to improve the dynamic range (Truong et al., 2001), stability under the acidic cytosolic environment (Miyawaki et al., 1999; Nagai et al., 2002, 2004), fluorophore maturation time (Nagai et al., 2002, 2004), the orientation between the two chromophores (Nagai et al., 2004), the binding kinetics between CaM and the CaM-binding peptide (Palmer et al., 2004), and the sensitivity to Ca^2+^ concentration (Palmer et al., 2006; Horikawa et al., 2010). More recently, Ding et al. developed a pair of Ca^2+^ (and caspase-3) FRET biosensors for simultaneous imaging of Ca^2+^ dynamics in the nucleus and the cytoplasm, when tagged with nuclear localization signal (NLS) and nuclear export signal (NES), respectively (Ding et al., 2011). Waldeck-Weiermair et al. (2012) developed a red-shifted Calemeon, called D1GO-Cam, and utilized it in conjunction with Fura-2, a ratiometric cytosolic Ca^2+^ dye (Grynkiewicz et al., 1985), to simultaneously monitor Ca^2+^ in cytoplasm and mitochondria. Due to heavy regulation of CaM in metabolic pathways, Cameleons suffer from apparent shortcomings in sensitivity for *in vivo* applications (Heim and Griesbeck, 2004). Therefore, TN-humTnC was developed for improved sensitivity and subcellular localization (Heim and Griesbeck, 2004). The sensitivity of this TN Ca^2+^ sensor has also been optimized through circular permutation of FPs (Mank et al., 2006), mutagenesis of TnC (Mank et al., 2008), the utilization of *Opsanus* TnC (Thestrup et al., 2014), and the adoption of yellow/orange (Su et al., 2012), and green/red color schemes (Fujii et al., 2013).

#### Myosin Light Chain Kinase (MLCK) FRET Biosensor

Myosin Light Chain Kinase catalyzes the phosphorylation of different sites on the regulatory light chain (RLC) of myosin II, thus either inhibiting or promoting its contractility depending on the phosphorylation of specific residues (Sellers et al., 1981; Ikebe et al., 1986). This constitutes a key regulator of cell contraction. MLCK activity itself is dependent on [Ca^2+^]_4_/CaM (Chew et al., 2002) and has been shown to regulate adhesion assembly (Webb et al., 2004). A FRET biosensor was developed, in which [Ca^2+^]_4_/CaM activates MLCK and affects binding between BFP and GFP to decrease FRET (Chew et al., 2002). The authors reported that prior to contraction, MLCK was transiently recruited to stress fibers and activated, suggesting a role of MLCK activation upstream to myosin contractility (Chew et al., 2002). MLCK activity was also observed in the protruding lamella of migrating cells and the spindle equator during cytokinesis (Chew et al., 2002). This MLCK FRET probe further allowed the discovery of RLC diphosphorylation and myosin contraction as the consequence of activation of endothelial MLCK during tumor intravasation (Khuon et al., 2010).

#### Epidermal Growth Factor Receptor (EGFR) FRET Biosensor

The EGFR family, a type of receptor tyrosine kinases (RTKs), is an integral part of the intracellular mechanotransduction pathways in response to external mechanical signals. E-cadherin-dependent EGFR localization and activation results in cell stiffening (Muhamed et al., 2016), regulation of tight junctions (Rübsam et al., 2017), and enhanced cell motility (Mateus et al., 2007). Upon activation, EGFR and its downstream signaling molecules, such as phospholipase C (PLC) and kinases in the inositol lipid pathway, are responsible for reorganizing cytoskeletal actin filaments (Payrastre et al., 1991; Rijken et al., 1991; van Bergen en Henegouwen et al., 1992; Yang et al., 1994) and generating local myosin contractile forces, likely through myosin light chain (MLC) phosphorylation by PLC-activated protein kinase C (PKC) (Iwabu et al., 2004). In rigid but not soft substrates, EGFR mediates ligand-independent rigidity sensing through Src-dependent phosphorylation (Saxena et al., 2017). In the osteogenic differentiation of human embryonic stem cells (hESCs), EGFR signaling has been suggested as a transducer from fibronectin fiber strain signals to biochemical cues (Li et al., 2013). A FRET biosensor contains the SH2 domain of Shc and EGFR phosphorylation peptide as sensing unit was first developed to monitor EGFR activity upon EGF stimulation (Ting et al., 2001). After that, (Offterdinger et al., 2004) designed a structurally disparate EGFR FRET reporter, named FLAME (fluorescent, linked autophosphorylation monitor for EGFR), comprised of EGFR, enhanced CFP (ECFP), a PTB domain, and citrine. Utilizing FLAME, the authors observed irresponsiveness of EGFR to EGF in the perinuclear region and accumulation of dephosphorylated EGFR in the perinuclear region after prolonged EGF treatment (Offterdinger et al., 2004). Another FRET reporter based on the SH2 domain of CrkII, named Picchu, was attached to the C-terminus of EGFR through amphipathic helixes to investigate the endocytosis of EGFR upon epidermal growth factor (EGF) stimulation (Itoh et al., 2005). Using this composite probe Picchu-Z, the authors observed that EGFR activity was maintained after endocytosis, and EGFR phosphorylation was lower at the perinuclear regions (Itoh et al., 2005), consistent with the prior study (Offterdinger et al., 2004).

#### Src FRET Biosensor

Src kinase plays a pivotal role in mechanical signal transduction. Src family kinases are coupled with T cell receptors (TCRs), B cell receptors (BCRs), RTKs (e.g., EGFR), G protein-coupled receptors (GPCRs), and CAMs (Thomas and Brugge, 1997). For instance, integrin β_3_ has been shown to directly activate Src by interaction mediated by its Src homology 3 (SH3) domain and Y418 phosphorylation, leading to Src substrate phosphorylation and downstream signaling (Arias-Salgado et al., 2003). Substrates of Src include but are not limited to focal adhesion kinase (FAK), PLC, and Shc. As such, Src activation presents profound effects on focal adhesion, migration, mitosis, and apoptosis (Thomas and Brugge, 1997).

The first Src kinase FRET biosensor developed consists of a CFP/citrine pair, an SH2 domain, and a Src substrate peptide obtained through library screening (Ting et al., 2001). This Src FRET reporter was not specific to Src, as considerable emission ratio change was also detected upon Lck, AbI, or EGFR phosphorylation (Ting et al., 2001). Therefore, by replacing the original substrate with one derived from p130Cas, (Wang et al., 2005) developed a Src-specific FRET sensor to visualize its activation dynamics in response to local mechanical loading in the human umbilical vein endothelial cells (HUVECs). A slow Src activation signal wave propagated along the plasma membrane of HUVECs after local bead loading, and disruption of actin filaments or microtubules eliminated long-range propagation of Src kinase activation signal (Wang et al., 2005). A subsequent study using this Src FRET biosensor found that fast Src signaling could be induced by stress but not EGF (Na et al., 2008). To date, alternative FP variants and color schemes have been adopted to improve FRET efficiency, including the use of EYFP variant YPet (Ouyang et al., 2008b), the mVenus/mKOκ (yellow/orange) pair (Su et al., 2012), the mTagBFP/sfGFP (blue/green) pair (Su et al., 2013), and most recently, the T-sapphire/stagRFP (green/red) pair (Mo et al., 2020).

#### G Protein-Coupled Receptors (GPCRs)

G protein-coupled receptors mediate signaling in hormonal and neural control of physiological functions, but their roles have also been implicated in mechanotransduction of cell volume change (Erickson et al., 2001), fluid shear stress (Das et al., 1997; Chachisvilis et al., 2006), traction forces (Marullo et al., 2020), and ECM interactions through the extracellular domains of GPCRs (Petersen et al., 2015; Scholz et al., 2015). A large archive of FRET probes has been developed to visualize the activities of Gα_i_, Gα_s_, and Gα_q_ subtypes. Due to the non-covalent nature of Gα/Gβγ interaction, all available GPCR FRET reporters are intermolecular. Bünemann et al. (2003) designed two bimolecular FRET reporters to monitor the dimerization of YFP-Gα_i_ with CFP-Gβ_1_ and CFP-Gγ_2_, respectively. GPCR activation leads to a reduction in FRET as a result of reduced affinity between Gα_i_ and Gβγ (Bünemann et al., 2003). Gibson and Gilman (2006) utilized a similar intermolecular FRET construct with the combination of different Gα_i_ and Gβ isoforms, revealing isotype coupling preferences. The brighter variants mTurquoise2 (mTq2) and cp173Venus were also utilized in Gα_i_ FRET probes for a higher dynamic range and photostability over long-term imaging (van Unen et al., 2015). Furthermore, FRET reporters were employed to characterize the kinetic parameters of G_q_-coupled M_1_ muscarinic receptor (M_1_R) activation, G_q_-mediated PLC activation, and PIP_2_ hydrolysis (Jensen et al., 2009). A bimolecular design similar to FRET probes for Gα_i_/ Gγ_2_ affinity was also developed to track Gα_q_ activation in living cells, with faster fluorophore maturation and larger dynamic range compared to the reporter developed by Adjobo-Hermans et al. (2011). The same intermolecular FRET strategy has been applied to a Gα_s_/Gβγ interaction probe (Hein et al., 2006).

#### RhoA (and Rho Family GTPases) FRET Biosensor

RhoA is a type of Rho-family small GTPases and has been implicated in cellular mechanical functions, including cytoskeleton remodeling, cell migration, focal adhesion, and phagocytosis (Hall, 2012). The application of FRET technology greatly advanced our understanding of Rho family GTPases in the cell cycle, migration, and plasticity. Two “Ras and interacting protein chimeric unit” (Raichu) FRET biosensors were developed to report RhoA-related activities which consist of a CFP/YFP FRET pair, the RhoA/GDP complex, and the RhoA-binding domain (RBD) of the effector protein (Yoshizaki et al., 2003). After phosphorylation by guanine nucleotide exchange factors (GEFs), RhoA/GTP binds to the RBD and leads to biosensor conformational change, thus increasing FRET (Yoshizaki et al., 2003). Therefore, Raichu-RhoA can be applied for the visualization of GEF activities in living cells. As Rho-specific guanine nucleotide dissociation inhibitors (RhoGDIs) prevent RhoA/GDP degradation and maintain its inactive state, Rho/GDP can be activated by GEFs once the interaction between RhoGDIs and RhoA/GDP changes due to post-translational modifications (PTMs) (Garcia-Mata et al., 2011). To visualize intracellular RhoA/GTP levels regulated by RhoGDIs, the second biosensor, Raichu-RBD, is comprised of an RBD from Rhotekin connected to one fluorophore at each side (Yoshizaki et al., 2003). Once RhoA/GDP is released from RhoGDI and becomes phosphorylated, the association between RhoA/GTP and the Rhotekin RBD will pull two fluorophores apart and decrease FRET (Yoshizaki et al., 2003). Using these two reporters, the authors were able to characterize the differential spatiotemporal dynamics of RhoA, Rac1 and Cdc42 activities in HeLa cells throughout mitosis (Yoshizaki et al., 2003). Another Cdc42/RhoGDI-based FRET biosensor based on a selective binding antenna was developed to monitor Cdc42-RhoGDI interaction, and similar strategies were suggested for studying RhoA-RhoGDI interaction (Hodgson et al., 2016). The standard CFP/YFP pair in the Raichu-RhoA construct was replaced by the brighter Clover and mRuby to enhance the photostability, independence of pH, and sensitivity of RhoA activity detection (Lam et al., 2012). The new Raichu-RhoA-CR was able to detect changes in RhoA activity within neuronal growth cones during ephrin stimulation (Lam et al., 2012).

Different biosensor structures have also been considered in RhoA FRET probe designs. Pertz et al. (2006) chose to put RhoA and RBD at the two terminals of the FRET reporter to monitor RhoA activity in different subcellular locations during cell migration and revealed the enrichment of RhoA activity at the protruding edges of randomly migrating mouse embryonic fibroblast (MEF) cells as well as polarized RhoA activity toward distal membrane sheets of peripheral ruffles upon both serum and platelet-derived growth factor (PDGF) induction. Interestingly, the authors demonstrated low RhoA activity in PDGF-induced MEF cell migration, supporting the antagonistic effect of PDGF-activated Rac on RhoA (Pertz et al., 2006). Further research utilizing this RhoA FRET biosensor revealed the spatiotemporal separation between Rac1 and (Cdc42) and RhoA at the leading edges of protruding MEFs (Machacek et al., 2009).

Due to the localization of RhoA kinase activity, several studies employed two-photon fluorescence lifetime imaging microscopy (2pFLIM) to increase the contrast of local fluorophore concentrations (Enrico et al., 2003). Murakoshi et al. (2011) constructed an intermolecular FRET biosensor by tagging RhoA with monomeric enhanced GFP (mEGFP) and Rhotekin RBD with one mCherry. In conjunction with a Cdc42 FRET reporter, this RhoA biosensor revealed disparate spatiotemporal patterns of RhoA and Cdc42 activation in dendritic spines of hippocampal CA1 pyramidal neurons during long-term potentiation (LTP) (Murakoshi et al., 2011). Subsequently, along with the Cdc42 reporter developed by Murakoshi et al. (2011) a red-shifted RhoA sensor was developed to simultaneously monitor their activities in dendritic spines undergoing synaptic plasticity (Laviv et al., 2016). Together with the green G-GECO1.1 Ca^2+^ indicator, a new intermolecular RhoA reporter based on RFPs uncovered the Ca^2+^ dependence of ATP-triggered RhoA activation in astrocytes (Nakahata et al., 2016).

#### Epigenetic FRET Biosensor

Traditionally regarded as storage for genetic information, the nucleus has been increasingly considered as a mechanosensor in recent years (Kirby and Lammerding, 2018). Force transmitted to the nucleus can alter the shape of the nuclear envelope, chromatin structure, and eventually transcriptional profile (Kirby and Lammerding, 2018). The impact of mechanical signals on epigenetics can be divided into two aspects: direct mechanical effects on the chromatin and biochemical signals converted from the force at the nuclear envelope. Force is transmitted across the cytoplasm via cytoskeleton (Maniotis et al., 1997), whereas force transmission through the nuclear envelope is mediated by the linker of nucleoskeleton and cytoskeleton (LINC) complex (Crisp and Burke, 2008; Chang et al., 2015). Chromatin may reorganize or decouple from nuclear lamina due to nucleocytoskeletal coupling. At the same time, possible mechanisms by which mechanical forces are converted into biochemical signals at the nuclear envelope include Ca^2+^ influx due to stretching-induced Ca^2+^ channel opening, transcription factor (TF) [such as MRTF, YAP (Dupont et al., 2011)] import and mRNA export through partial opening of nuclear pore complex (NPC), protein unfolding and phosphorylation in response to mechanical force, and altered histone acetylation and methylation patterns (Wang et al., 2009; Maurer and Lammerding, 2019). For example, fluid shear stress can lead to enhanced histone deacetylase (HDAC) activity, inducing H3K14ac, and H3K27ac (Illi et al., 2003, 2008; Fish et al., 2005; Chen et al., 2010; He et al., 2019). A recent study has shown that the rapid up-regulation of mechanoresponsive genes depends on H3K9me3 demethylation (Sun et al., 2020). However, whether chromatin reorganization is downstream of cytoplasmic mechanotransduction or due to direct force on the nucleus, as well as detailed mechanisms regarding how force causes protein phosphorylation and how chromatin stretching activates specific mechanosensitive genes are currently unclear.

Currently, FRET has been applied to detect histone H3 and H4 PTMs. The earliest FRET H3K9me3 and H3K27me3 reporters were each based on a partial H3 peptide and a methyllysine binding domain (Lin et al., 2004). Subsequently, an H3S28p FRET reporter with the same structure was developed with a different H3 peptide and a phosphoserine-binding domain (Lin and Ting, 2004). A more recent version of the H3K9me3 FRET probe adopted a different structure and utilized the entire H3 protein so that this probe can be integrated into chromosomes to directly quantify the histone modification (Peng et al., 2018). Furthermore, a series of FRET biosensors based on the BRD4 domain were developed to monitor H3K9ac, H3K14ac (Nakaoka et al., 2016), H4K5ac, H4K8ac (Sasaki et al., 2009), and H4K12ac (Ito et al., 2011).

## FRET-Based High-Throughput Drug Screening

Since there is more and more evidence showing that mechanobiological factors play crucial roles in disease, drugs targeting mechanobiological signaling pathways would be promising. Unlike conventional biochemical assay-based drug-screening platforms, FRET-based high-throughput screening (HTS) platform has a high signal-noise ratio and spatio-temporal resolution (Rothman et al., 2005; Marine et al., 2006), and enables the measurement of molecular events that occur relatively fast in single live cells. In the majority of FRET biosensor designs, FRET can be altered, either induced or abolished, by catalytic activities, conformational changes caused by protein-protein interactions, or direct force. These FRET signals can be applied for high-throughput screening (HTS) of enzymes and small molecule drugs in a therapeutic setting. As such, FRET-based screening platforms have shown great potential in evaluating therapeutic-relevant drugs (Lu and Wang, 2010; Mizutani et al., 2010).

Förster or fluorescence resonance energy transfer method combined with subcellular imaging has been successfully applied in versatile HTS assays for different targets and diseases over the past decade. Time resolved (TR)-FRET-based HTS in which FRET ratio change is triggered by protein-protein interactions has been applied to the discovery of small-molecule inhibitors for Httex1 aggregation in Huntington’s disease (Lo et al., 2020), calcium release by endoplasmic reticulum due to familial AD-linked presenilin 1 mutations (FAD-PS1) in Alzheimer’s disease (Honarnejad et al., 2013), binding of anthrax protective antigen and capillary morphogenesis gene 2 (CMG2) protein in angiogenetic cancers and anthrax intoxication (Rogers et al., 2012), I&κB kinase β (IKKβ) and non-canonical ubiquitin-conjugating enzyme UBC13 in inflammatory diseases (Oh et al., 2010; Madiraju et al., 2011), and histone methylation activity of lysine demethylase 1 (LSD1) and Jumonji C domain-containing oxygenase D2C (JMJD2C) (Yu et al., 2011). Recent studies employed TR-FRET-based HTS to screen and successfully identify small molecule drugs that affect cardiac sarcoplasmic reticulum Ca-ATPase (SERCA2a) structure (Gruber et al., 2014; Schaaf et al., 2016) or increase the affinity between SERCA2a and phospholamban (PLB), presenting a potential therapeutic solution to cardiac contractile dysfunction due to deficient Ca^2+^ transport (Stroik et al., 2018). Furthermore, cleavage-based FRET HTS has been applied to the screening of enzymatic activities or small molecules that can affect the activity of particular enzymes with high consistency. For example, the proteolysis activity of Atg4A and Atg4B could be measured in a high-throughput setting with high reproducibility (Li et al., 2012). A similar cleavage-based FRET assay measuring ganglioside-processing enzymes was also shown to provide consistent results among different samples (Yang et al., 2015).

Förster or fluorescence resonance energy transfer-based HTS can also be employed to optimize FRET biosensors, and in combination with flow cytometry, to promote screening efficiency. For example, the linker length of a caspase-3 FRET biosensor SCAT3 was optimized through HTS (Nagai and Miyawaki, 2004). Similarly, the FPs used in the biosensor were optimized through directed evolution and HTS by fluorescence-activated cell sorting (FACS) to achieve a higher dynamic range so that the optimized caspase-3 FRET biosensor can directly measure the activity of caspase-3 in flow cytometry, allowing the identification of caspase-3-dependent apoptotic cells via HTS (Nguyen and Daugherty, 2005). The combination of FRET and FACS has also allowed HTS of the interactions between HIV proteins and cellular proteins (Banning et al., 2010), and between adaptor proteins and GTPases (Thyrock et al., 2010). FRET-assisted FACS further enabled the HTS of a serine protease OmpT variant library (Olsen et al., 2000). Thus, FRET assays can be applied for drug screening in a high throughput manner. Developing HTS platforms should significantly advance mechanopharmacological studies.

## Limitations, Challenges of FRET Biosensors and Future Perspectives

Applications of FRET in live-cell imaging have significantly advanced our understanding of how cells sense and respond to the mechanical microenvironment. However, the majority of the works were mainly performed *in vitro*. At the current stage, several mouse models with FRET biosensors were successfully generated, such as the mouse strains with Src (Nobis et al., 2013), calcium (Atkin et al., 2009), MLCK (Isotani et al., 2004), and RhoA (Nobis et al., 2017) biosensors. Visualization of mechanotransduction using mouse models should further expand the boundary of our understanding of mechanobiology in a more biological and complicated microenvironment. Furthermore, although several different signaling molecules were identified to be involved in mechanotransduction, how these different molecules or signaling pathways interplay with each other during the expeditious and dynamic mechanotransduction processes remains to be elucidated. The spatiotemporal visualization of different signaling molecules by combining multiple FRET biosensors with distinct colors should be helpful to solve this problem.

Although FRET biosensors have revolutionized the imaging of molecular signals in live cells with high spatiotemporal resolutions, the limitations in sensitivity and specificity have hindered their broader applications. Another limitation on the application of FRET biosensors is the efficient delivery of genetically-encoded constructs into cells, especially for hard-transfect primary cells, and the subsequent establishment of cell lines. The aforementioned QD FRET biosensor, which bypasses the delivery of genetically encoded FP-based biosensors, serves as a desirable alternative to overcome such challenge (Chung et al., 2015). While handling adherent cells during imaging is relatively simple, it is very challenging to analyze suspension cells with imaging systems, since they float freely in media and the focus or the observation field can easily be lost over time, especially at high magnification. Thus, due to practical considerations, the application of FRET biosensors is mainly for adherent cell imaging. Thus, future optimizations of FRET biosensors with high efficiency is highly needed. We believe that the development of high throughput, high-efficiency screening platform, together with automatic, high throughput imaging analysis and quantification platform [e.g., Fluocell (Qin et al., 2019)], should greatly benefit the FRET-based mechanistic study and screening.

The impact of biomechanical factors on drug actions is being highlighted lately as an emerging consideration. Although the impact of mechanics on cellular responses is well recognized and continuously raising attentions, there are limited studies on how mechanobiological factors influence pharmacological responsiveness (Krishnan et al., 2016). Mechanopharmacological screening should provide novel mechanistic insights into the drug response of cells under the different microenvironment. In fact, studies on the liver tumor *in vitro* models demonstrate that stiffness priming of stromal cells could influence the chemo-resistance of tumor tissue, highlighting the importance of mechanopharmacological screening (Zhu et al., 2017). Due to the importance of mechanobiological factors in disease progression, efforts were made in developing HTS-mechanopharmacological assays. For example, the contractile force screening (CFS) platform was developed based upon straightforward measurement of cellular contractile force and is suitable for scaling to medium to high throughput (Park et al., 2015). Besides, fluorescently labeled elastomeric contractible surfaces (FLECS) embedded in elastomers enables the single-cell force measurements in a high throughput manner (Pushkarsky et al., 2018). Using micro- and nanotopography, a 96-well plate platform was developed to screen topographies and drug-topography combinations that have short- and long-term effects on T cell activation and proliferation (Hu et al., 2016). At tissue level, high throughput fibrotic microniches (FμNs) were developed for measuring the contraction force in tissues and screening drugs that target fibrosis expression, which demonstrated excellent predictability of the drug efficiency *in vivo* (Liu et al., 2017). However, current available mechanopharmacological assays mainly quantify the cell phenotype, which may be influenced by multiple different signaling pathways. To our knowledge, the current FRET-HTS has not been developed for mechanopharmacological screening of specific molecular events. As such, the successful combination of FRET technology with HTS-mechanopharmacological bioassays should provide a more powerful screening platform with high specificity that enables the screening of molecules with novel mechanism of action during mechotransduction.

## Author Contributions

All authors listed have made a substantial, direct and intellectual contribution to the work, and approved it for publication.

## Conflict of Interest

YW was a scientific co-founder of Cell E&G and Acoustic Cell Therapy Inc. However, this does not affect the content of this article. The remaining authors declare that the research was conducted in the absence of any commercial or financial relationships that could be construed as a potential conflict of interest.
